# Self-healing effects in a semi-ordered liquid for stable electronic conversion of high-energy radiation

**DOI:** 10.1038/s41598-018-30815-w

**Published:** 2018-08-17

**Authors:** Bradley R. Nullmeyer, Jae W. Kwon, J. David Robertson, Alexander Y. Garnov

**Affiliations:** 1 Department of Electrical Engineering & Computer Science, University of Missouri-Columbia, Columbia, Missouri 65211 USA; 2 Department of Chemistry, University of Missouri-Columbia, Columbia, Missouri 65211 USA; 3 University of Missouri Research Reactor (MURR), University of Missouri-Columbia, Columbia, Missouri 65211 USA

## Abstract

Radiation damage in solid-state semiconductors has, until now, placed strict limitations on the acceptable decay energies of radioisotopes in radiovoltaic cells. Relegation to low-energy beta-emitting isotopes has minimized the power output from these devices and limited the technology’s ability to deliver greater energy densities and longer lifetimes than conventional batteries. We demonstrate the self-healing abilities of a liquid-phase semiconducting alloy which can withstand high-energy alpha radiation. Neutron diffraction of liquid selenium-sulfur shows the liquid phase repairing damage sustained in the irradiation of the solid phase. This self-healing behavior results in long-lived power output in a liquid selenium-sulfur alphavoltaic cell. To the best of our knowledge, this marks the only successful demonstration of resistance to high-energy radiation (>500 keV) in a semiconducting material. This new robustness can potentially allow increases to the available energy density in radiovoltaic cells near 1000 times the current state of the art.

## Introduction

Over the past century, nuclear radiation has been explored for a wide range of applications due to its ability to deliver large amounts of energy from a small amount of material. However, the high energy emissions from radioactive isotopes are damaging to atomic and molecular structures, and are well-known for their destructive effects. In certain applications such as therapeutic radiation, this destruction is the desired outcome. In other uses, however, radiation-induced damage is simply a byproduct of the intended effect and can become a significant or even prohibitive problem. This obstacle can only be truly overcome by materials which preserve their desired properties (mechanical, electrical, chemical, etc.) upon bombardment with ionizing radiation. However, such robustness has not been presented for most behaviors and applications. Such is the case for radiovoltaic power, which has been theoretically proposed as a revolutionary improvement in sourcing power to portable, distant, and inaccessible systems, but has been unable to surmount the technical challenge of radiation-induced destruction.

Historically, chemical batteries have been the dominant method of supplying portable power. Due to large size, heavy weight, and frequent recharging requirements of conventional batteries, scientists have been searching for a new paradigm of long lasting power sources using radioisotopes^1–7^. These alternative energy sources have high energy density (approximately 10^6^ times greater than that of electrochemical reactants), and theoretically long lifetimes (potentially hundreds of years), allowing a miniscule source to power dependent technology for decades. Thus, radioisotopes have long been considered promising candidates for the future of power sources in a variety of demanding applications, especially deep space exploration and other remote operations such as unmanned lighthouses and distant sensors^8–12^. However, radioisotope thermoelectric generators (RTGs), such as those used in the NASA Voyager I and II probes are difficult to miniaturize due to thermal management components such as heat distribution blocks, cooling tubes, and copious thermal insulation, which are not easily fabricated on the microscale^10–13^. Moreover, the emerging Stirling radioisotope generators require similar components, as well as moving parts^14,15^. These thermal and mechanical components of the existing state of the art also add significant weight to a system, which critically affects spacecraft engineering, operability, and cost. Conversely, the direct conversion process in a radiovoltaic cell requires only a semiconductor and thin-film electrodes, allowing operation in a miniscule package.

While radiovoltaic cells present many advantages over other technologies in theory, their progression has been slow. Lattice dislocations in crystalline semiconductors caused by the impact of high energy radiation degrade power conversion over time and eventually cause failure^2,16–19^. For this reason, many efforts have selectively utilized low energy beta emitters such as ^3^H (E_avg_ = 5.9 keV) and ^63^Ni (E_avg_ = 17.4 keV) in pursuit of long-lasting devices^20–22^. As an alternative strategy, others have utilized wide-bandgap semiconductors, which have greater threshold displacement energies and are less susceptible to radiation damage. For example, a 2006 study reported a SiC cell exhibiting strong resistance to radiation from ^33^P, which has an average beta energy of 77 keV, with a lifetime of over three months^23^. However, the use of higher energy beta-emitting isotopes has remained out of reach for direct conversion using semiconductors because of the radiation damage imparted by high-energy beta particles and the external radiation field generated outside of the cell from over-penetrating beta and Bremsstrahlung radiation.

**Figure 1:**
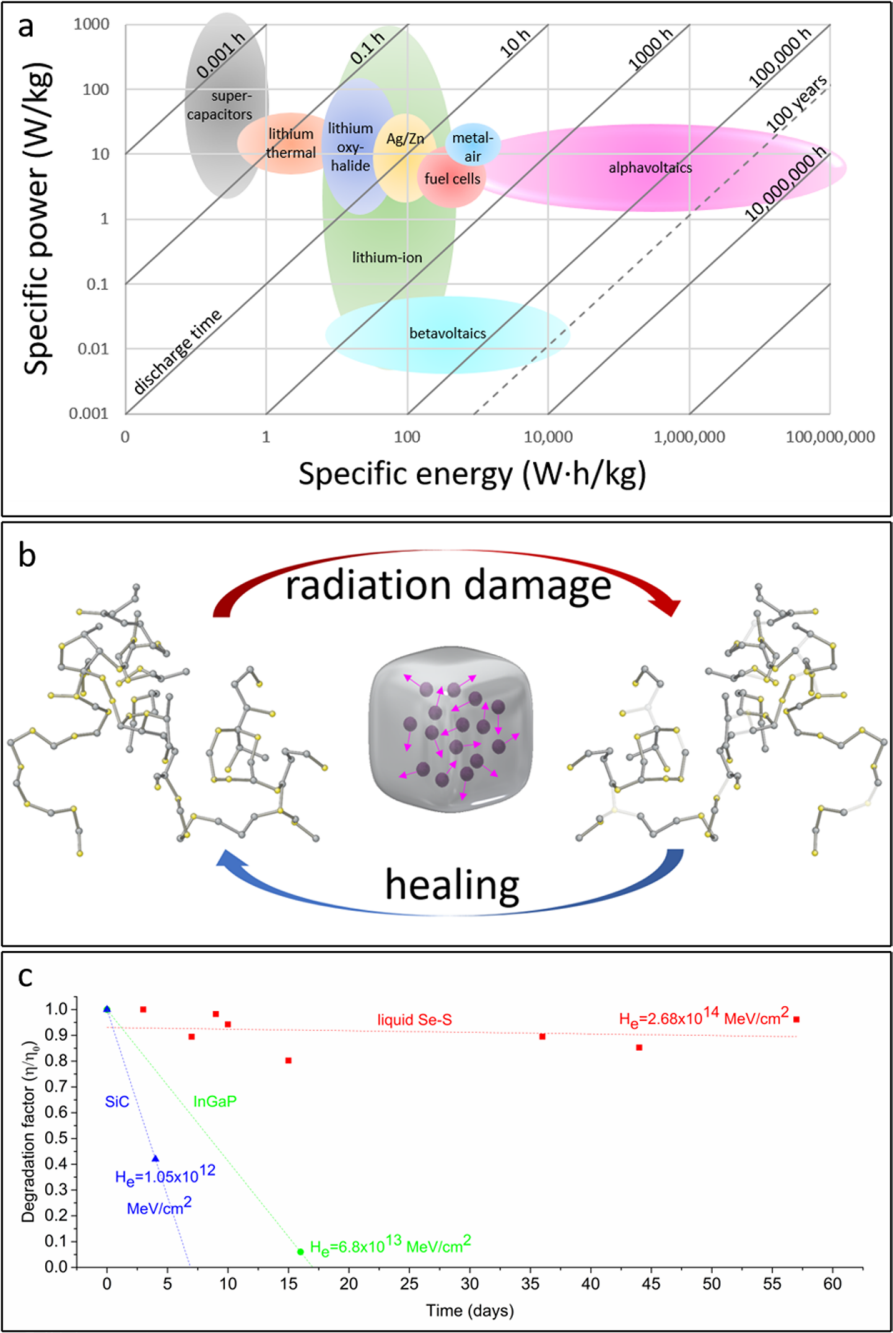
An overview of the semi-ordered liquid semiconductor as an alphavoltaic material. **(a)** The power and energy densities of alphavoltaic cells far exceed those of other technologies, with extraordinarily long discharge times. Alpha-emitting isotopes are more power-dense than beta-emitters by 2–3 orders of magnitude and lifetimes are extended by a 432-year half-life for ^241^Am. **(b)** Radiation-induced damage to a semi-ordered liquid semiconductor initially occurs when bonds are broken or atoms are ejected upon recoil from collisions with alpha particles (subsequent defects arise from free radicals as well). The material maintains an equilibrium structure by constantly re-arranging itself to eliminate metastable defects. The continuous nature of this process allows liquid Se-S to self-heal radiation damage. In this experiment, polonium-210 alpha-emitting radionuclides were infused throughout liquid Se-S to maximize absorption of the emitted radiation for radiovoltaic power generation. **(c)** The extrapolated lifetime for a liquid Se-S alphavoltaic is virtually failure-less compared to extrapolation of SiC and InGaP lifetimes based on published experimental data. The Se-S cell reported herein remained near the initial efficiency for 57 days of testing, with a total alpha energy fluence which was much greater than the severely damaging fluences for the previously reported solid-state cells. The energy fluence is noted at the final data point of each device, calculated from the time, isotope decay energy, and activity per unit area.

As shown in Fig. 1a, functional alphavoltaic cells could exhibit energy densities and lifetimes which are orders of magnitude greater than current technology, including betavoltaics. While alpha radiation has significantly greater energy than beta radiation (often by two orders of magnitude), it also results in more severe lattice displacement damage in materials due to high momentum from the large mass of alpha particles (approximately 7300 times more massive than beta particles). Previous reports have shown a strong correlation between non-ionizing energy loss (NIEL) of a particle and displacement damage in solids^24^. This relationship emphasizes the extreme difference between the interactions of alpha and beta particles with matter and the damage incurred. For example, the NIEL for a 5 MeV alpha particle is approximately 10^4^ times that of a beta particle of the same energy in silicon. Considering a more realistic beta energy of 100 keV, the NIEL of a 5 MeV alpha particle is approximately 10^5^ times greater. Thus, alpha radiation is much more damaging and has often been ignored as a real possibility in radiovoltaic power generation. Previous efforts have been primarily focused on wide-bandgap solid-state materials, which have shown greater beta radiation resistance than silicon, but ultimately, the resulting cells exhibited rapid degradation in the presence of higher energy alpha radiation. In 1996, rapidly decreasing power output from a SiC cell irradiated by ^241^Am was reported, followed by similar results from another effort in 2011^16,17^. In 2005, similar degradation was confirmed in InGaP *n-i-p-i* structures^18^. Another InGaP effort in 2006 reported similar drastic declines in performance over time^19^. The alpha radiation fluxes used in these experiments span a wide range of values, with every instance being on the order of microcuries per square centimeter. The degradation of these solid-state materials in relation to radiation fluence and time is illustrated in Fig. 1c.

To our knowledge, no evidence of resistance to damage from high-energy radiation in a semiconductor material has been observed in any previous effort (as indicated in Table 1). A lack of resistance to such high-energy emissions diminishes the capability of radiovoltaic cells by limiting the isotope selection and available energy, and the resulting restriction to low-energy beta emissions ensures extremely low power output. Consequently, the proposed applications for radiovoltaics have shifted from widespread deployment and system-level power to merely niche micropower devices, and the possibilities for more powerful radiovoltaics are generally overlooked.

**Table 1:** A summary of previously reported radiovoltaics.

Ref.	semi-conductor	radioisotope	power	total efficiency	rapid failure
^1*^	Si	^90^Sr/^90^Y (β^−^, 196/940 keV)	800 nW	0.4%	Yes
^3^	Si	^147^Pm (β^−^, 61.8 keV)	50 μW	unreported	unreported
^7^	Si	^147^Pm (β^−^, 61.8 keV)	612 nW	1.02%	unreported
^20^	Si	^63^Ni (β^−^, 17 keV)	0.32 nW	0.31%	unreported
^21^	a-Si	^3^H (β^−^, 5.7 keV)	130 nW	0.013%	unreported
^9^	Se-S	^35^S (β^−^, 49 keV)	687 nW	7.05%	unreported
^22^	SiC	^63^Ni (β^−^, 17 keV)	30.2 pW	unreported	unreported
^23^	SiC	^33^P (β^−^, 85 keV)	580 nW	0.56%	No
^17^	SiC	^63^Ni (β^−^, 17 keV)	2.04 nW	0.5%	No
^17*^	SiC	^241^Am (α, 5.5 MeV)	1.25 nW	0.1%	Yes
^16*^	SiC	^241^Am (α, 5.5 MeV)	15 nW	0.009%	Yes
^18*^	InGaP	^210^Po (α, 5.3 MeV)	30 nW	0.19%	Yes
^19*^	InGaP	^210^Po (α, 5.3 MeV)	0.0068 nW	0.04%	Yes
^19*^	InGaP	^241^Am (α, 5.5 MeV)	0.0504 nW	0.46%	Yes

*Every device which included high energy radiation exhibited rapid failure.

Conceding that high-energy radiation will ultimately result in damage to any material, an alternative “self-healing” material may instead be used to avert the accumulation of radiation-induced defects over time. Certain *liquid* semiconductors have simple polymeric structures which may be easily repaired by natural atomic motions and interactions. Unfortunately, most liquid semiconductors exhibit metallic electronic structure, with Fermi energies near or within the conduction band. Conversely, molten selenium and sulfur both exhibit insulating behavior, with Fermi energies well separated from conducting bands^25–27^. The use of a Se-S mixture has been previously reported in low-energy ^35^S betavoltaic cells, and has been suggested to be potentially radiation resistant, but this claim has not yet been substantiated^28^.

The atomic structures of liquid selenium and sulfur have been estimated theoretically and experimentally^29–35^. Neutron and x-ray scattering investigations have supported the original theoretical claims that both materials are composed of chain-like molecules, with the average chain length being dependent on thermodynamic conditions. This implies that chains can break and re-combine as the material heats and cools, with the structure remaining nearly constant at a given temperature. Moreover, analysis has found that frequent thermally-driven collisions in liquid selenium result in both new bond formation and chain breaking to reach lower-energy configurations, resulting in a thermodynamically-regulated equilibrium structure^36^. Thus, although the structure will be locally affected by incident ionizing radiation, the system is likely to continue in its natural, constant re-arrangement of bonds, resulting in a self-healing mechanism. Likewise, since molecules in liquids are free to move, the concept of dislocation or displacement of atoms is null. This self-healing equilibrium behavior of liquid Se-S can potentially solve the problem of radiation damage, which has been the most troublesome issue for radiovoltaic cells since their inception. Herein, we present strong evidence for the self-healing ability of liquid Se-S from neutron scattering investigations of the irradiated structure. This self-healing ability is demonstrated by long-lived power generation in a liquid Se-S alphavoltaic cell.

## Results

### Neutron diffraction study of radiation damage and self-healing effects in Se-S

**Figure 2:**
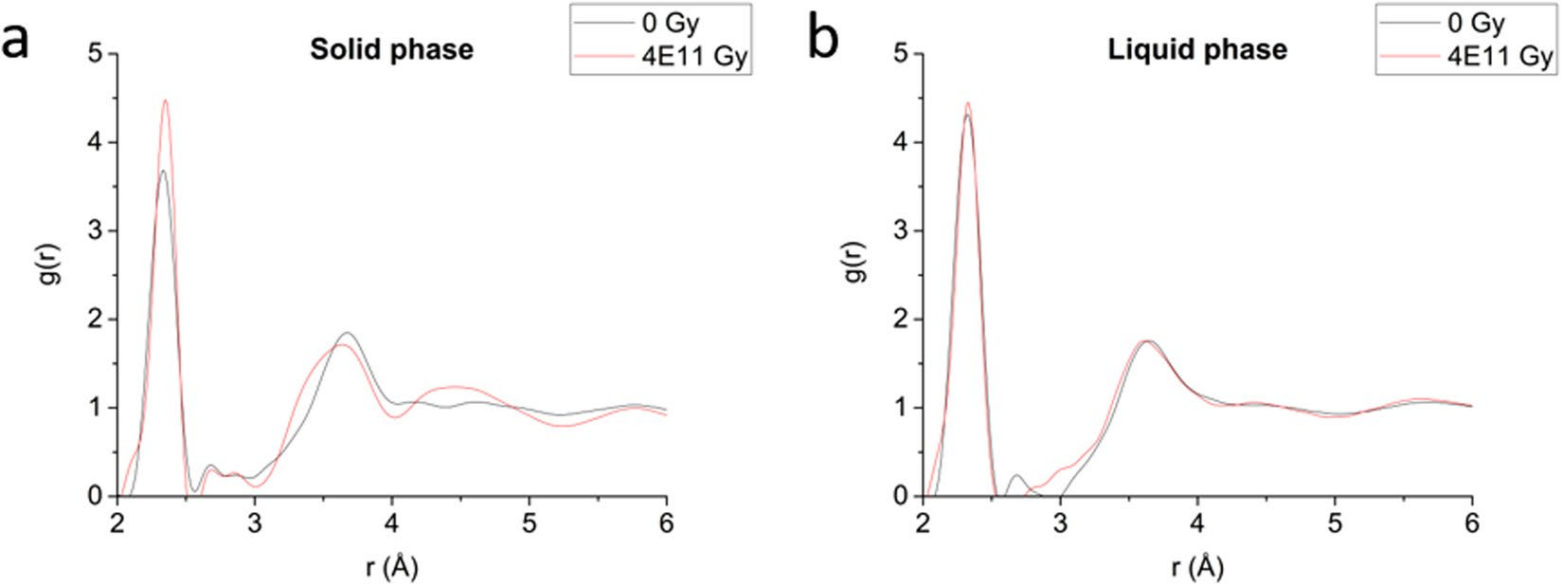
Pair correlation functions of Se-S in the solid and liquid phases. **(a)** The irradiated sample shows a considerably greater first peak than the non-irradiated sample in the solid phase. **(b)** This difference vanishes in the liquid phase.

Stability of the Se-S atomic structure and the associated self-healing effects were studied using neutron diffraction and compared in both the solid and liquid phases before and after exposure to varying doses of high-energy, high-linear-energy-transfer radiation. To investigate radiation damage on non-radioactive samples, a proton beam was used to irradiate Se-S samples to doses of 4 × 10^7^ and 4 × 10^11^ Gy. These doses correspond to the energy deposited in a 10-mg Se-S alphavoltaic material by one year of constant alpha particle emissions from 250 μCi and 2.5 Ci of 5.3 MeV alpha particles. The irradiated samples, along with a non-irradiated control, were then analyzed using the Nanoscale-Ordered Materials Diffractometer (NOMAD) at Oak Ridge National Laboratory (Oak Ridge, TN). The irradiated samples were first analyzed at *T* = 293 K in the solid phase, and again at *T* = 500 K in the liquid phase. Figure 2 shows a comparison of the pair correlation function of un-irradiated and highly-irradiated Se-S in both the solid and liquid phases. Among the solid samples, the height of the first peak increases upon irradiation. This peak is associated with the primary bond length, with a greater area under the peak implying a greater number of bonds. This would indicate that the selenium-sulfur chains are cross-linked or the average chain length gets longer as a result of irradiation in the solid phase. It should be noted that cross-linking may occur naturally at higher temperatures as well, implying that the addition of energy to the system not only breaks existing bonds, but partially reconfigures the overall structure^37^. Moreover, photonically induced transitions to a more cross-linked structure have been reported as well^38^. Thus, the increased coordination is consistent with previous estimations of energetically-induced structural changes. Conversely, the unirradiated and irradiated liquid samples exhibited virtually no variation in the height of the first peak on the pair correlation function.

### Reverse Monte Carlo modeling of Se-S neutron diffraction

**Figure 3:**
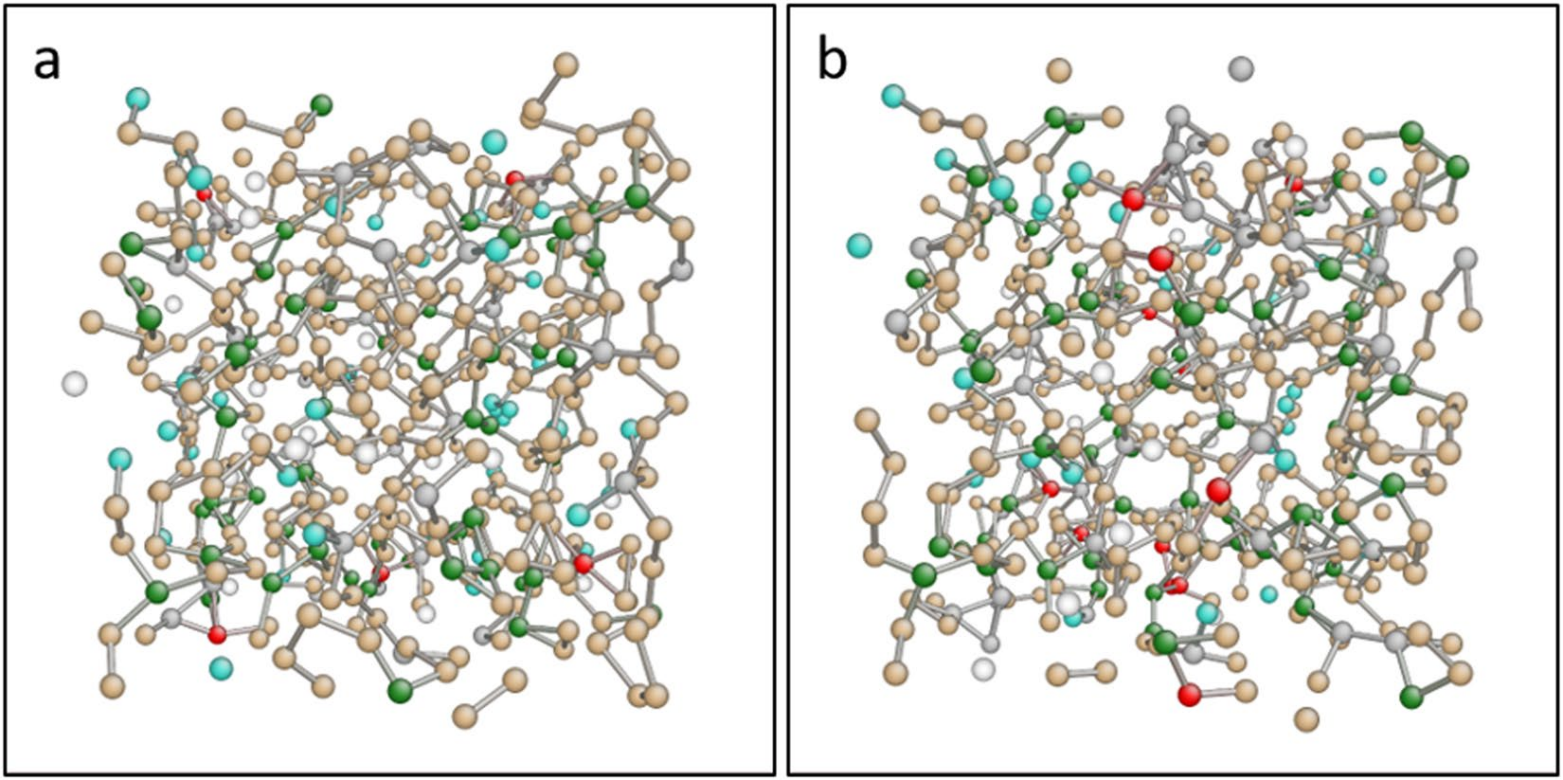
Reverse Monte Carlo models of the un-irradiated Se-S structure in the (**a**) solid phase and (**b**) liquid phase (500 K). Both phases are structured as a network of interconnected chains, while the liquid is more highly coordinated. The atoms are color-coded by their coordination number: white = 0, turquoise = 1, beige = 2, green = 3, gray = 4, red = 5.

Reverse Monte Carlo (RMC) analysis of the neutron diffraction data was performed using RMCProfile software, version 6.5.2 and the AtomEye program included in the RMCProfile package^39,40^. This computational approach generates three-dimensional molecular models from experimental scattering data. Figure S3 shows a comparison of the experimental pair correlation functions and those of the RMC-generated models for each scattering measurement. The RMC-generated Se-S configurations are web-like for both solid and liquid phases, with short chains cross-linked by atoms of 3+ coordination, as shown in Fig. 3. Although previous efforts to interpret scattering data of liquid selenium and liquid sulfur have concluded chain/ring structures for both materials, molecular dynamics modelling efforts have generated more complex structures for liquid selenium, similar to the model proposed here^41,42^.

Given the theoretical chain-like structure for liquid selenium and liquid sulfur, two-fold coordination was assumed to be the standard configuration, with any other coordinates being considered “defects.” One- and three-fold coordinates are believed to be the most common defects in liquid selenium, and their presence has been shown to affect the density of states, altering the material’s band structure^43^.

### Radiation induced defects in solid Se-S

**Figure 4:**
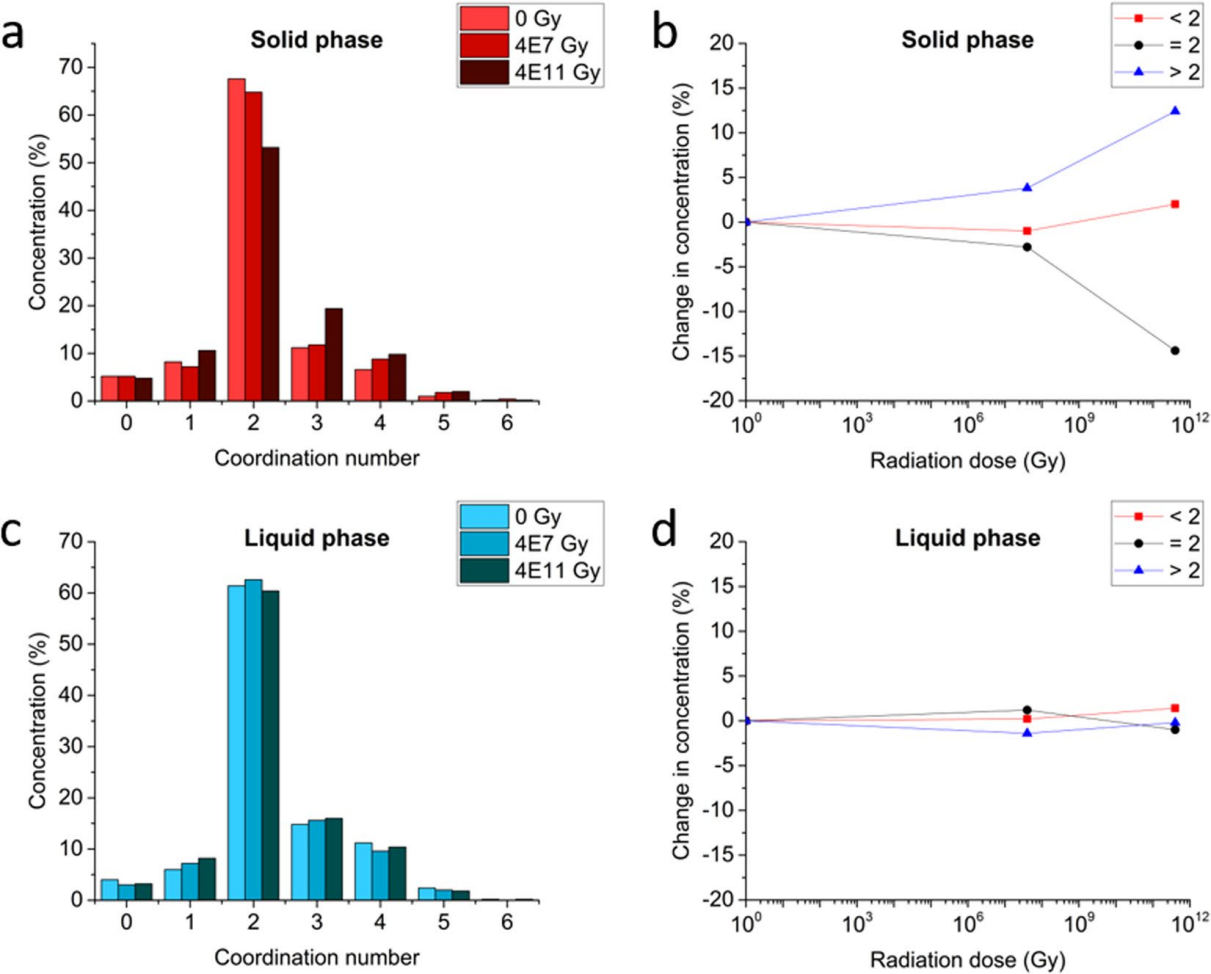
The coordination statistics in Se-S, obtained from Reverse Monte Carlo modeling of neutron diffraction results. **(a)** The coordination statistics of the solid exhibit radiation-induced changes correlated with the dose. **(b)** Upon irradiation in the solid phase, the non-defective two-fold coordination decreases in concentration, while highly coordinated defects emerge. **(c)** The liquid-phase samples exhibit minimal differences at varying radiation doses. **(d)** Minor fluctuations in the liquid’s coordination are not correlated with the absorbed radiation dose.

The RMC configurations for each solid and liquid sample reveal a remarkable disparity between the radiation effects in solid Se-S compared to the liquid. The non-irradiated solid sample is estimated to have an average coordination number of 2.1, which is increased by radiation to 2.2 and 2.3 in the samples exposed to 4 × 10^7^ and 4 × 10^11^ Gy, respectively. Closer analysis of the changes in coordination show that the concentration of two-fold coordinates is reduced from 67.6% to 64.8% and 53.2% in the irradiated samples. The concentration of one-fold defects fluctuates, while the concentration of three-fold defects progressively increases with the absorbed dose from an initial 11.2% to an eventual 19.4%. The number of four-fold defects also increases progressively from 6.6% to an eventual 9.8%. Given the limitations of the RMC process and a fixed two-fold coordination constraint imposed on the model, it is reasonable to interpret the structure by assessing the net concentration of defects less than two (0- and 1-fold) and more than two (3+). The number of atoms with coordination less than two fluctuates without exhibiting substantial increases, while the concentration of 3+ coordination increases from 19.0% in the un-irradiated sample to 21.8% at 4 × 10^7^ Gy and 31.4% at 4 × 10^11^ Gy. These coordination statistics are displayed graphically in Fig. 4, and are also denoted in Table S1.

It is likely that the bombardment of selenium chains severs bonds, leading to unstable electronic states which re-form at higher coordination. This concept is supported by a reported molecular dynamics (MD) simulation of photon-induced excitation in a selenium chain molecule^36^. The lowest unoccupied molecular orbital (LUMO) of the selenium chain is of anti-bonding character in the ground state. However, when an electron is excited across the band-gap, the amplitude of the LUMO wave function becomes intense near the Se-Se bond, and the chain is broken to stabilize the wave function. The shortened chains feature new states which are located inside the original bandgap with intense wave function amplitudes near the ends of the chain, and are capable of re-bonding with neighboring chains, even in hypervalent configurations. This effect has previously been reported for photonically-excited solid selenium, which exhibited a 6–7% increase in the average coordination number^38^. The changes have been indicated by modeling to result from the formation of 3-, 4-, and 5-fold defects. In this proton irradiation experiment, a 9.5% increase in the average coordination number was observed from an absorbed dose of 4 × 10^11^ Gy.

Previous efforts to characterize defects in liquid selenium have identified coordination defects as electronic states which disrupt the band structure of the system^36,43^. Analysis of the localized density of states for each defect type revealed that 1-fold coordination defects create states within the bandgap near the valence band, and 3-fold defects create states near the conduction band. As the material develops a more highly coordinated structure, the upper half of the bandgap fills with electronic states, dragging the conduction band to lower energies. Thus, the energy of the conduction band approaches the Fermi level, leading to a more n-type material with a narrower bandgap.

### Healing radiation damage in the liquid phase

In contrast to the solid diffraction results, the Reverse Monte Carlo models for the liquid-phase Se-S show minimal variation among the sample with no clear dose-related trends, as shown in Fig. 4c,d. The average coordination number for the un-irradiated sample was 2.3 for all three samples (un-irradiated, 4 × 10^7^ Gy, and 4 × 10^11^ Gy). Based on this evidence, it appears that the liquid phase of the Se-S composite is un-altered by the absorbed high-energy radiation. All three samples exhibit the same liquid structure, regardless of the radiation history; thus, the material exhibits a self-healing ability in the liquid phase. This is consistent with the results of previously reported time-step MD modelling efforts, which show that the structure of liquid selenium undergoes frequent re-arrangement due to self-diffusion^43,44^. Thus, continuous evolution of the local structure may be the mechanism by which defects are eliminated in the Se-S system.

### Long lifetime in a liquid Se-S alphavoltaic

**Figure 5:**
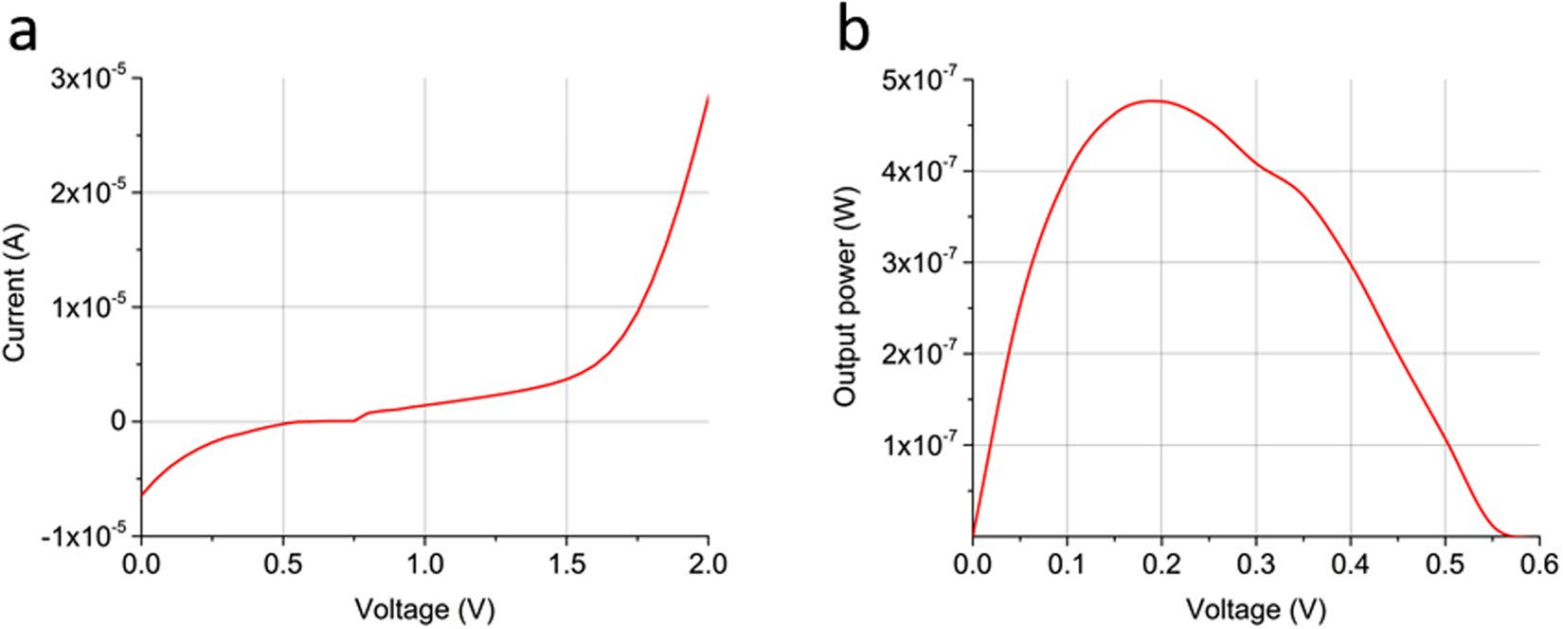
Electronic performance of the Se-S alphavoltaic. **(a)** Single-measurement I-V curve. **(b)** Associated power output. The cell produced an average power output of 429 nW from 277 µCi of ^210^Po.

The self-healing behavior of liquid selenium-sulfur was demonstrated by long-lived alphavoltaic performance in a cell consisting of liquid Se-S and radionuclides sealed in a micro-reservoir between gold and aluminum electrodes. The gold was coated with 277 μCi of polonium-210 (^210^Po) in the form of dried PoCl_4_, which was drop-cast from a purified solution of polonium in dilute hydrochloric acid. The fabrication, and assembly processes for the device are illustrated in Figs. S1 and S2. Polonium-210 was selected due to its high specific activity and chemical compatibility with selenium and sulfur. The isotope monoenergetically emits 5.3 MeV alpha particles, with a half-life of 138 days. As shown in Fig. 1c, the alphavoltaic performance was assessed for 57 days using I-V characterization. The cell operated at an average total efficiency of 5.5% over the experiment’s long duration, with an average power output of 429 nW. The open circuit voltage was 0.6 V and the short circuit current was approximately 6.5 μA. A representative I-V measurement from the cell is shown in Fig. 5. Variation in the power output was carefully observed, and can be attributed to unstable contact at the liquid/electrode interfaces inside the cell (as the melted material did not fill the entire reservoir). The liquid-semiconductor-based alphavoltaic did not exhibit a rapid decrease in power output, which has been the clear indication of radiation damage in previous efforts^16–19^.

## Discussion

From the experiments and analyses presented herein, it is clear that the liquid phase of Se-S is capable of healing remarkable amounts of radiation-induced damage. Vast changes to the atomic structure were observed in radiation-exposed samples at room temperature, with the growth of defects correlated to the radiation dose. Moreover, the mechanism of radiation damage was determined to be an overall increase in atomic coordination of the sulfur and selenium atoms. These alterations to the coordination statistics are theoretically linked to the Fermi level and the electronic characteristics of Se-S. In the liquid phase, this damage to the atomic structure was “healed,” as exhibited by minimal variation in the atomic structure across all samples, with no correlation to radiation dose. Thus, it is concluded that the primary mechanism for electronic degradation in the Se-S alphavoltaic system is eliminated in the liquid phase. This characteristic was demonstrated by the stable, long-term power output of the studied Se-S/^210^Po alphavoltaic cell.

Certainly, possibilities for other issues warrant further investigation. Selenization and sulfurization of electrodes due to recoil implantation, as well as doping of the liquid semiconductor by particles sputtered from solid electrodes during alpha bombardment are possible long-term effects which extend beyond the scope of this work and provoke further experimentation and analysis. During the study period presented in this article, electronic degradation of the cell was not observed despite the possibilities for these phenomena. This newfound radiation-resistance of a liquid semiconductor re-invigorates the concept of practical radiovoltaic power. While radiovoltaic cells had been previously limited to low-energy beta emitters, this work introduces a potential pathway to reliably harvest high energy emissions, including alpha particles in the MeV range. By transitioning to higher-energy isotopes, the power densities of radiovoltaic cells can be expanded roughly 1000 times compared to the current state of the art, allowing deployment in a variety of applications for which radiovoltaic power has been greatly anticipated, but historically insufficient. Moreover, this work encourages further study on molecular self-healing mechanisms for degradation-resistant materials.

## Methods

### Experimental design

Two experiments make up the findings presented in this manuscript. The first experiment was performed with the goal of fabricating and evaluating a liquid Se-S alphavoltaic cell, with the hypothesis that the cell would exhibit long-term stability and be free of radiation-induced degradation. To accomplish this, a device was fabricated using microfabrication techniques and loaded with Se-S powder and an alpha-emitting ^210^Po radioisotope. Heat was then applied to melt the Se-S and seal the cell. The completed device comprises two glass substrates with an SU-8 spacer, with each substrate being coated with an electrode material. One electrode is a gold Ohmic contact, while the other is an aluminum Schottky contact. The Se-S converting semiconductor is sandwiched between the two electrodes and surrounded radially by the SU-8 spacer. The second experiment was a neutron diffraction analysis of irradiated Se-S material. Se-S was irradiated by a proton beam using a cyclotron system and subsequently analyzed in the solid and liquid phases using neutron diffraction. The resulting scattering measurements were interpreted with using the Reverse Monte Carlo simulation technique to better understand the spatial distribution of atoms in a three-dimensional system.

### Preparation of Se-S powder for use in the alphavoltaic

The Se-S powder was prepared by mixing the two materials in a 3:2 molar ratio, followed by partial dissolution in a CS_2_ solvent and evaporation at room temperature. The dried material was then pulverized into a powder.

### Preparation of ^210^Po solution

The radioisotope ^210^Po was chosen for this experiment due to its high activity density and chemical compatibility with selenium and sulfur. The isotope decays by alpha emission at an energy of 5.3 MeV, with a very rare gamma emission. Its relatively short half-life of 138 days is consistent with an experimental observation timeframe, and allows substantial radioactivity with a small amount of material. Thus, the cell can be loaded with a small molar concentration of the radioisotope to avoid disruption of its electronic characteristics.

The ^210^Po used in this experiment was manufactured via neutron irradiation of ^210^Bi. A bismuth oxide target (natural bismuth being 100% ^209^Bi) was irradiated in the University of Missouri Research Reactor (MURR), initiating neutron capture which transmuted a portion of the material to ^210^Bi via the ^209^Bi(n,γ)^210^Bi reaction. The ^210^Bi then underwent β^−^ decay with a half-life of 5.01 days, resulting in ^210^Po radionuclides within the bulk bismuth oxide. The target was then dissolved in nitric acid and liquid-liquid extraction was used to separate the polonium nuclides from bismuth using tributyl-phosphate (TBP) and dibutyl-ether (DBE) before introducing the polonium solute into concentrated hydrochloric acid. The hydrochloric acid was then vaporized and the remaining polonium chloride was dissolved again using dilute hydrochloric acid to minimize the amount of chloride salts which would eventually enter the cell. This dilute hydrochloric acid/^210^Po solution would eventually be drop-cast and vaporized on the alphavoltaic cell’s gold electrode, as a final means of introducing the ^210^Po into the device.

### Alphavoltaic cell fabrication procedure

The fabrication of the alphavoltaic cell began with the sputter deposition of metallic films on “1 × 1.5” borosilicate glass substrates (Borofloat®, SI Howard Glass). A gold electrode was deposited on one substrate, with an underlying chromium adhesion layer. The other substrate was coated on both sides with an aluminum electrode. Each electrode was patterned by photolithography, while a small piece of Kapton tape was used to preserve an interconnected electrode across each face of the glass substrate. A spin-coater was used to deposit 10 μm-thick SU-8 films on each piece, which were subsequently patterned by photolithography, resulting in a circular reservoir over each electrode. The two pieces were then heated at 95 °C for 12 hours to fully cure the SU-8. When the two pieces are joined during the loading procedure, a 20 μm-thick reservoir, enclosed by the electrodes, is formed. This fabrication process is illustrated in Fig. S1.

### Alphavoltaic cell loading and bonding procedure

The cells were loaded and bonded by hand in a nitrogen-filled glove box. The bottom half of the device was placed on a hotplate and heated to 120 °C. The PoCl_4_ aqueous solution was extracted into a micro-pipette and drop-cast onto the surface of the gold electrode. After complete evaporation of the aqueous solution, Se-S powder was placed on the gold electrode and covered by the top half of the device. A “1 × 1.5” borosilicate protective glass cover spacer was then placed on top, followed by a 1-kg weight. The hotplate was then set to 285 °C for twelve minutes, then powered off. Several hours later, with the system cooled, low-viscosity epoxy was injected around the edges of the device to create an air-tight seal. This loading process is illustrated in Fig. S1.

### Preparation and irradiation of Se-S for radiation damage study

To analyze the effects of high-LET radiation on solid and liquid Se-S, the material was irradiated to varying doses by a proton beam. This procedure began with the preparation of a bulk Se-S mixture by melting the two elements in a crucible. Selenium was heated to 300 °C on a hotplate while covered. Sulfur was then introduced to the crucible at high temperature, while the molten mixture was stirred and then quickly covered. The material was then cooled to room temperature before being pulverized with a pestle and ground into a fine powder. The powder was loaded in small batches into a specially designed sample holder which was fitted for the University of Missouri Research Reactor (MURR) cyclotron beam.

### Neutron scattering data processing

The Se-S samples were analyzed using the Nanoscale-ordered Materials Diffractometer (NOMAD) instrument at Oak Ridge National Laboratory (Oak Ridge, TN). The neutron scattering intensity from the samples was measured first in the solid phase at room temperature and then again in the liquid phase at an elevated temperature of 500 K. The system’s automated sample-changing cryostat, was used to heat each sample and control its temperature during measurement, with a jet of hot gas acting as the heat source.

The neutron scattering intensity data was reduced by the NOMAD instrument’s autoreduction process. This yields the structure factor S(Q) and the pair correlation function g(r), which are related by a Fourier transform:

$$g(r)-1=\frac{1}{2{\pi }^{2}r\rho {\sum }^{}{b}^{2}}{\int }_{Qmin}^{Qmax}\,(S(Q)-1)Qsin(Qr)dQ$$

During the reduction process, the g(r) distribution function was smoothed using the Lorch function. The samples were first analyzed at *T* = 293 K the solid phase, and again at *T* = 500 K in the liquid phase.

### The Reverse Monte Carlo process

The Reverse Monte Carlo method is an algorithm which begins with a configuration of particles and attempts to move them to match a set of structural input data. RMCProfile 6.5.2 was used to generate Reverse Monte Carlo models of selenium-sulfur, starting from a randomly distributed set of 300 selenium atoms and 200 sulfur atoms. The initial configuration was generated using the RMCProfile *dwbuild* script, with a number density of 0.037.

For a semi-ordered material, it is critical to guide the RMC process with proper constraints to obtain a realistic model^39^. In the discussed simulations, a fixed coordination constraint was used to guide the system toward a structure of purely two-fold coordination, as suggested by the selenium and sulfur theoretical models and previous interpretations of diffraction data^29–32^. Although the software sought such a structure, it was not possible to fully reconcile the scattering data with purely two-fold coordination, thus resulting in the coordination statistics presented in Table S1.

### Data availability

The structural data presented herein is available in the Supplementary Material associated with this article. Additional data Table S1 contains the pair correlation functions for solid and liquid Se-S at varying radiation doses, obtained by neutron diffraction. Additional data Table S2 contains the RMC-generated configuration files for the Se-S structure.

## Electronic supplementary material

Supplementary Information
