# Supplementary material for: Self-healing effects in a semi-ordered liquid for stable electronic conversion of high-energy radiation
Source: Sci Rep. 2018 Aug 17;8:12404. doi: 10.1038/s41598-018-30815-w (PMC6098062; doi:10.1038/s41598-018-30815-w)
Supplement: Supplementary file 1 — Supplementary Information [file 41598_2018_30815_MOESM1_ESM.pdf]

Self-healing effects in a semi-ordered liquid for stable electronic conversion of high-energy radiation

Bradley R. Nullmeyer, Jae W. Kwon, J. David Robertson, Alexander Y. Garnov

Supplementary Information

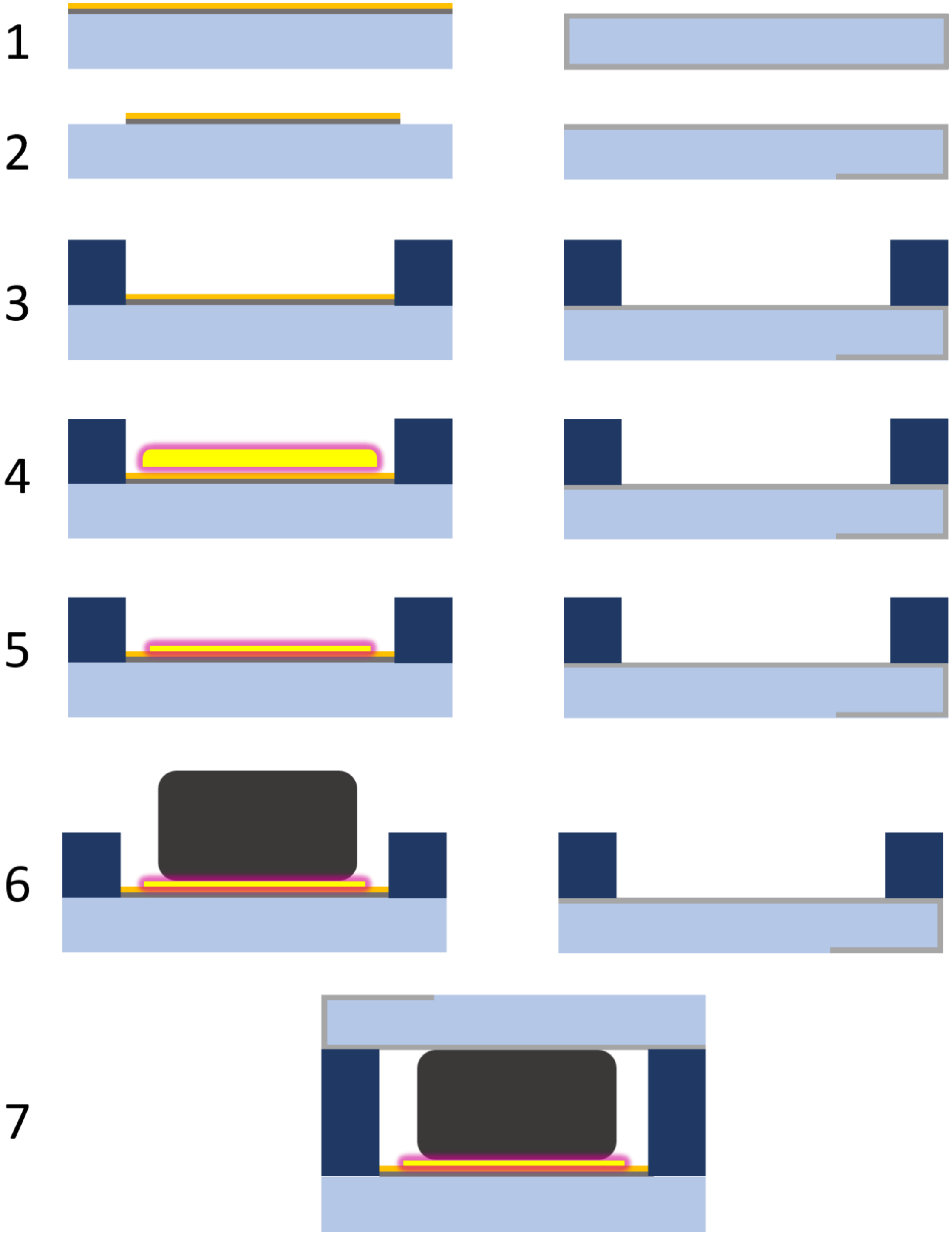

Fig. S1. Fabrication and loading process for the alphavoltaic battery: 1) Sputter electrodes on glass substrates; 2) pattern electrodes with photolithography; 3) spin-coat and photopattern SU-8 reservoir on both substrates; 4) drop-cast $^{210}Po/HCl$ solution on the gold electrode; 5) vaporize liquid, leaving only the dried $^{210}PoCl_4$ salt on the gold surface; 6) place Se-S in the device by hand; 7) assemble two pieces to seal the device.

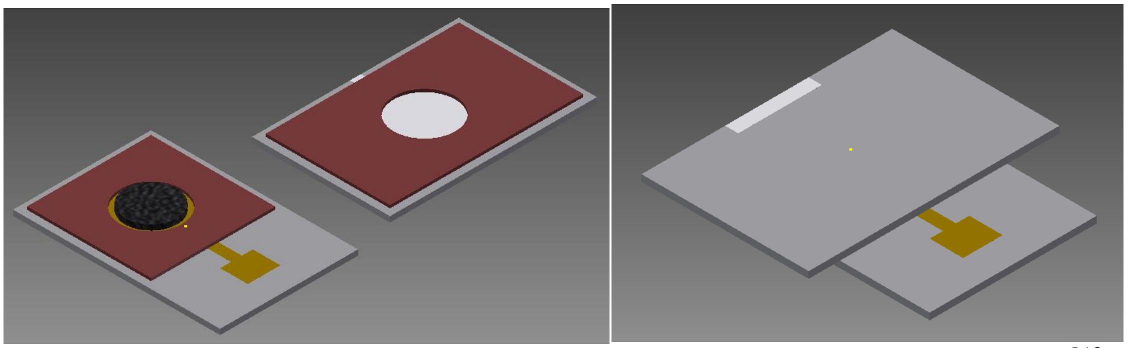

Fig. S2. Illustration of the alphavoltaic cell. After the gold electrode is loaded with ^{210}Po and Se-S, it is enclosed by the aluminum-coated cover piece.

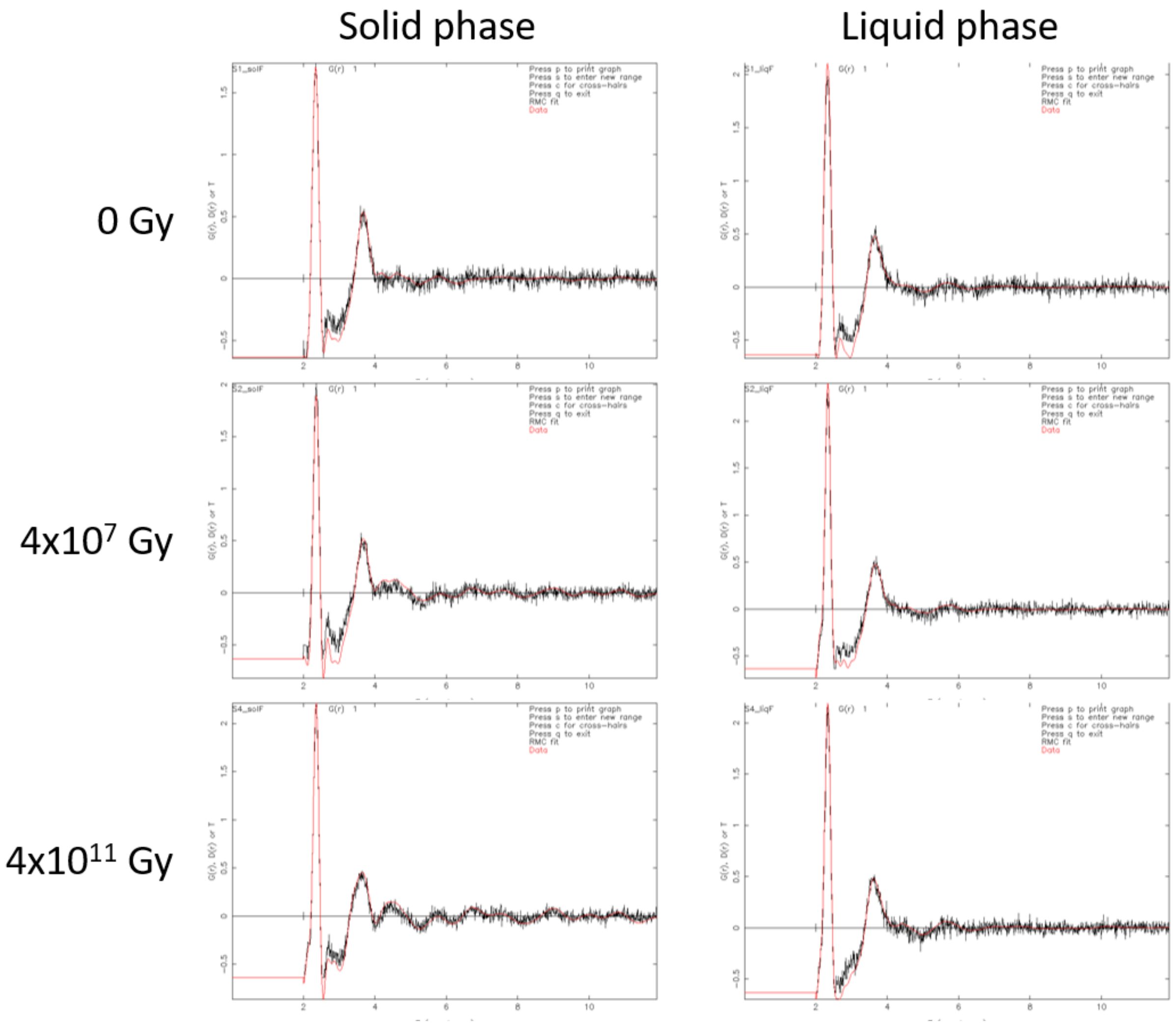

Fig. S3. Pair correlation functions and the fit of RMC-generated models. Each model had with a χ^2 value less than 1 for goodness-of-fit to the experimental data.

Table S1. Coordination number statistics generated by the RMC method

CN	Content in control (0 Gy) solid	Content in irradiated (10^7 Gy) solid	Content in irradiated (10^{11} Gy) solid	Content in control (0 Gy) liquid	Content in irradiated (10^{11} Gy) liquid	Content in irradiated (10^{11} Gy) liquid
0	5.2 %	5.2 %	5.2 %	4.0 %	3 %	3.2 %
1	8.2 %	7.2 %	10.6 %	6.0 %	7.2 %	8.2 %
2	67.6 %	64.8 %	53.2 %	61.4 %	62.6 %	60.4 %
3	11.2 %	11.8 %	19.4 %	14.8 %	15.6 %	16.0 %
4	6.6 %	8.8 %	9.8 %	11.2 %	9.6 %	10.4 %
5	1.0 %	1.8 %	2.0 %	2.4 %	2.0 %	1.8 %
6	0.2 %	0.4 %	0.2 %	0.2 %	0.0 %	0.2 %
AVG. CN	2.1	2.2	2.3	2.3	2.3	2.3

Additional data table S1 (separate file)
The g(r) pair correlation functions for each sample, as measured by neutron diffraction. Six input files are labelled according to the corresponding radiation dose and phase. For example, 4E11Gy_solid_gr.dat contains the g(r) data for the Se-S sample which was irradiated to $4x10^{11}$ Gy, analyzed in the solid phase.

Additional data table S2 (separate file)
The configuration files which were generated by the RMC simulations for each sample in the solid and liquid phases. These files include the three-dimensional coordinates for the atoms in each model. Six configuration files are labelled according to the corresponding radiation dose and phase. For example, 4E11Gy_solid.cfg contains the configuration data for the Se-S sample which was irradiated to $4x10^{11}$ Gy, analyzed in the solid phase.
